# Demystifying the Functional Role of Nuclear Receptors in Esophageal Cancer

**DOI:** 10.3390/ijms231810952

**Published:** 2022-09-19

**Authors:** Sujitha Jayaprakash, Mangala Hegde, Sosmitha Girisa, Mohammed S. Alqahtani, Mohamed Abbas, E. Hui Clarissa Lee, Kenneth Chun-Hong Yap, Gautam Sethi, Alan Prem Kumar, Ajaikumar B. Kunnumakkara

**Affiliations:** 1 Cancer Biology Laboratory, Department of Biosciences and Bioengineering, Indian Institute of Technology (IIT) Guwahati, Guwahati 781039, Assam, India; 2 Radiological Sciences Department, College of Applied Medical Sciences, King Khalid University, Abha 61421, Saudi Arabia; 3 BioImaging Unit, Space Research Centre, Michael Atiyah Building, University of Leicester, Leicester LE1 7RH, UK; 4 Electrical Engineering Department, College of Engineering, King Khalid University, Abha 61421, Saudi Arabia; 5 Electronics and Communications Department, College of Engineering, Delta University for Science and Technology, Gamasa 35712, Egypt; 6 Department of Pharmacology, Yong Loo Lin School of Medicine, National University of Singapore, Singapore 117600, Singapore; 7 NUS Center for Cancer Research, Yong Loo Lin School of Medicine, National University of Singapore, Singapore 119228, Singapore

**Keywords:** esophageal cancer, nuclear receptors, agonists, antagonists, biomarkers, treatment

## Abstract

Esophageal cancer (EC), an aggressive and poorly understood disease, is one of the top causes of cancer-related fatalities. GLOBOCAN 2020 reports that there are 544,076 deaths and 604,100 new cases expected worldwide. Even though there are various advancements in treatment procedures, this cancer has been reported as one of the most difficult cancers to cure, and to increase patient survival; treatment targets still need to be established. Nuclear receptors (NRs) are a type of transcription factor, which has a key role in several biological processes such as reproduction, development, cellular differentiation, stress response, immunity, metabolism, lipids, and drugs, and are essential regulators of several diseases, including cancer. Numerous studies have demonstrated the importance of NRs in tumor immunology and proved the well-known roles of multiple NRs in modulating proliferation, differentiation, and apoptosis. There are surplus of studies conducted on NRs and their implications in EC, but only a few studies have demonstrated the diagnostic and prognostic potential of NRs. Therefore, there is still a paucity of the role of NRs and different ways to target them in EC cells to stop them from spreading malignancy. This review emphasizes the significance of NRs in EC by discussing their diverse agonists as well as antagonists and their response to tumor progression. Additionally, we emphasize NRs’ potential to serve as a novel therapeutic target and their capacity to treat and prevent EC.

## 1. Introduction

Esophageal cancer (EC) is an aggressive and poorly understood disease that remains one of the leading causes of cancer-related deaths around the world [1,2]. This cancer is fundamentally resistant to systemic therapy due to morphological, molecular, and etiological heterogeneity [3]. Even though there are various advancements in treatment procedures, this cancer has been reported as one of the most difficult cancer to cure, and a favorable prognosis is only possible in the pilot stages [4]. EC is one of the most common types of cancer in people; GLOBOCAN 2020 estimated 604,100 new cases and 544,076 fatal cases worldwide [5]. Individuals with EC have dismal 5-year overall survival (OS) rates [3]. The incident rate of EC varies a lot depending on their location [1,2,6,7]. Squamous cell carcinoma (SCC), adenocarcinoma (AC), and other subtypes of EC are histologically classified, and more than 95 percent of esophageal malignancies are squamous cell carcinoma and adenocarcinoma [1,2,6,7]. The histological subtype of EC with the highest incidence is esophageal squamous cell carcinoma (ESCC). The histology of human ESCC follows a stepwise pattern of dysplasia, hyperplasia, and SCC, and it originates from precancerous lesions [8].

Common risk factors for ESCC include smoking tobacco, excessive alcohol consumption, and chronic inflammation, which have been proven to have some synergistic impact on the development of EC [8,9]. Moreover, dietary variables, genetic factors, microbes, and other environmental factors may all have a role in the disease’s etiopathogenesis [8,9]. The geographical disparities show that genetic factors, ethnicity, and lifestyle play a significant influence in the development of ESCC, as demonstrated by the high incidence rates of ESCC in Southern Europe, Southern and Eastern Africa, and East Asia compared to North America and other regions of Europe [2]. Adenocarcinoma is the most predominant EC in Europe and North America [7]. Esophageal adenocarcinoma (EAC) and gastric cardiac adenocarcinoma (GCA) are two types of ACs that arise at the junction of the distal esophagus and the proximal stomach [10]. EAC is becoming a common source of mortality and morbidity, where patients who are suffering from this disease have a 17 percent survival rate, as this cancer is detected in later stages following the local invasion and/or metastasis [7]. Smoking, obesity, alcohol consumption, nutritional deficit, genetic factors, Helicobacter pylori infection, Barrett’s esophagus (BE), and chronic gastroesophageal reflux disease (GERD) are the main risk factors for EAC [1].

BE, a metaplastic alteration of the typical squamous mucosa of the esophagus into a columnar lining, is the solely recognized precursor for EAC [11]. Moreover, the presence of BE is linked to an elevated risk of EC by 30 to 40 folds [11]. Currently, only 5% of individuals with EAC have the precancer diagnosis of BE [2,12,13]. Although EC is asymptomatic in its initial stages; dysphagia, unintended weight loss, nausea, anorexia, abdominal pain, odynophagia, and bloating are the most common presenting symptoms at the early stage, which makes the diagnosis difficult [7,14,15]. Patients with EC have had a better general prognosis in recent decades because of surgical and medicinal advances, although overall survival remains dismal [16]. EC treatment is complicated and varies between nations and centers [17]. However, there is a need for a more effective biomarker for the treatment of EC because of its complications and aggressiveness.

The most utilized therapeutic modality before esophagectomy is radiochemotherapy [17,18]. However, the development of chemoresistance, significant adverse medication reactions, and high treatment costs present the main therapy hurdles for this disease at an advanced stage [19]. Therefore, the development of medications that are safe, effective, and economical continues to be a challenge in the field of EC research.

**Figure 1:**
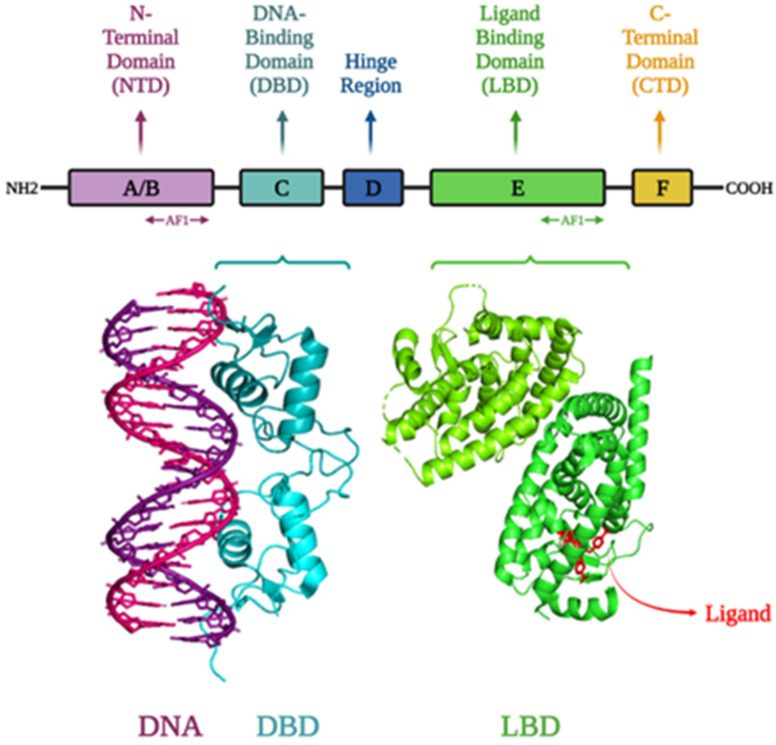
Structure of different domains of NRs: N-terminal domain, DNA-binding domain, hinge, ligand/hormone binding domain, and C-terminal domain; Structure of the progesterone receptor-DNA complex at a resolution of 2.50 Å (PDB ID: 2C7A); Crystal structure of the complex between PPAR gamma ligand binding domain and the ligand AM-879 at a resolution of 2.69 Å (PDB ID: 6AN1). Visualization of the structures was performed using PyMOL and saved them as .jpg files [44,45,46,47,48].

Many human diseases, such as metabolic disorders, autoimmune diseases, and cancer, are linked to dysregulated tissue metabolism and inflammation [20,21,22,23,24,25]. The transcriptional changes in metabolic and immunological cells in response to pathogenic stimuli received from the microenvironment are largely associated with transcription factor (TFs) abnormalities [20]. Nuclear receptors (NRs) are a type of TFs found in cell nuclei that bind with certain ligands, respond to hormones, and regulate several physiological processes in the cell [26,27]. They also act as a key regulator in reproduction, development, cellular differentiation, stress response, immunity and metabolism [28,29,30,31]. Moreover, these receptors have an impact on a wide range of genes and are involved in a complex network of signaling pathways [27,32,33]. NRs are essential for controlling several disease states, including diabetes, obesity, atherosclerosis, and cancer, in addition to normal homeostatic and metabolic processes [30]. Depending on the cell type, numerous coregulators and NR functions have been studied together in various contexts [34]. There are 48 TFs in the human NR family, including receptors for lipophilic vitamins, cholesterol metabolites such as retinoic acid and oxysterols, thyroid hormones, steroid hormones, and fatty acids, whose dysregulation frequently results in disease states [28,35,36,37]. Most NRs are notable therapeutic targets because of the ability of small compounds to specifically activate or inactivate them [28,36,38,39]. The classification of NRs’ superfamily was based on evolutionary sequence conservation observed among various receptors. These receptors are thought to have split into seven subfamilies thus far [27,28]. Adopted-Orphan-Receptors (Lipid-sensors and Enigmatic-Orphans), Endocrine-Receptors, and Orphan-Receptors are the three main groups of the 48 members of the superfamily of NRs [40]. The first category consists of receptors with a high affinity for their ligands, such as seco-steroidal receptors (VDR and RARs) and steroidal receptors (AR and ER). The FXRs, LXRs, and PPARs have modest binding affinities for a wider variety of lipophilic compounds and are included in the second category. NR4A1/NUR77 and ERRs are examples of the final group that have not yet been identified as ligands or do not have a ligand binding domain [40,41]. The structure is common by all members of the NR superfamily consists of a variable N-terminal region, a ligand-independent transactivation function (AF-1) domain, and a highly conserved DNA-binding domain (DBD), which binds to particular DNA sequences known as hormone response elements (HREs). These proteins’ C-terminal region contains the dimerization interface, ligand-dependent activation function (AF-2), and ligand binding domain (LBD) (Figure 1). The activation state of the NR is altered by ligand interaction, which either activates or inactivates its transcriptional output [42,43]. The majority of NR ligands are tiny, lipophilic molecules that may easily diffuse across the plasma membrane of cells and bind their associated receptors [28]. Most of the NRs’ activities can be regulated by endogenous and exogenous substances, such as metabolites, steroid hormones, and synthetic compounds [20].

Moreover, NRs have been intensively studied in cancer biology because they have shown tremendous promise as new therapeutic targets for various cancer types due to their high druggability and actionability qualities [26]. As a result, around 16 percent of FDA-approved medications now target NRs, emphasizing the relevance of NRs in human disease [49]. Moreover, NRs have become promising targets for anticancer drug development due to their impact on a variety of cancer-related processes (e.g., tumor initiation and therapeutic response) [49]. NRs are adaptable cellular ‘sensors’ because of their ability to respond fast and dynamically to numerous developmental and environmental signals by altering gene programs. As a result, NRs have long been used as biomarkers for the classification of a variety of solid tumors, including breast and prostate malignancies, as well as hormone therapy targets [50].

According to the growing number of data, NRs function as a modulator of signaling that connects the inflammatory response to the development and progression of cancer, and they control particular genes with tumor-suppressive or cancer-causing properties [34,51,52,53,54,55,56,57]. For example, the impact of steroid hormones on prostate cancer has been demonstrated by one of the earliest research projects on NRs. They demonstrated the activation of prostate cancer by androgen injections [58]. A surfeit number of investigations have demonstrated the significant role of NRs in tumor immunology and proved the roles of multiple NRs in modulating proliferation, differentiation, and apoptosis suggest that NRs and their ligands have direct antitumor effects on cancer cells and can be used as cancer immunotherapeutic targets [26,30]. It is well established that various NRs such as androgen receptors (ARs), estrogen receptors (ERs), farnesoid X receptors (FXRs), peroxisome proliferator-activated receptor *γ* (PPAR*γ*), retinoic acid receptors (RARs), retinoid X receptors (RXRs), and vitamin D receptor (VDR), which shows association with cancer development [28,36,59,60]. Surprisingly, important roles of AR and ER in the etiological factors of breast cancer and prostate cancer have been discovered, respectively. AR expression in epithelial cells is thought to cause prostate cancer, and this cell type’s role in the development of the disease is significant. Moreover, prostate cancer is assumed to be caused by the excessive stimulation of the ER system because of increased ER levels [34]. Moreover, the oncogenic role of AR, ER, GR, PPAR and VDR in tumor-supporting cells is well characterized [31]. Further, estrogen and progesterone receptor expression remain therapeutically significant in predicting prognosis and deciding therapy options for breast cancer [36]. An in vitro study has demonstrated that NR4A1, which is an orphan receptor, is overexpressed in pancreatic cancer and regulates cancer cell survival and death [8]. According to a tissue-specific and FXR-null mice study, it has been shown that FXR has been linked to the development of gastrointestinal and liver malignancies and operates as a suppressor of hepatocellular cancer, primarily via maintaining BA homeostasis [61]. All other NRs, including RARs, RXRs, PPARs, GRs, and PRs, have also been extensively studied as cancer-therapeutic targets [62,63]. It was intriguing to discover that numerous NRs have been associated with EC and can be targeted as a new therapeutic target to prevent the disease’s progression. For instance, in a study, inhibition of FXR by FXR shRNA or guggulsterone decreased EC tumor development and growth in nude mice xenografts, as well as decreased tumor cell viability and incited apoptosis in vitro. Therefore, it is clear that EC can be effectively controlled by inhibiting FXR expression or activity and could be a therapeutic target [64].

There are a surplus number of studies conducted on NRs and their implications in EC, but there is still a lot to learn about how to target EC cells and stop them from spreading malignancy. The significance of NRs involved in EC using agonists and antagonists and their response to tumor growth is highlighted by this review. Synthetic medications have continued to exhibit severe side effects and the development of chemoresistance despite recent advancements in treatment approaches, restricting their applicability [65,66,67,68]. Because they have few adverse effects, phytochemicals are increasingly being used [69,70,71,72]. Furthermore, research over the previous four decades has illuminated the therapeutic and cancer-prevention potential of natural compounds as well as their underlying mechanisms of action [35,73,74,75,76,77,78,79]. Some natural substances have been discovered to be more effective in treating various types of cancer than the various modern chemotherapies [80,81,82,83,84,85,86,87,88]. Numerous studies have discovered the huge potential of a variety of non-toxic, multi-targeted natural compounds/agents in overcoming drug resistance in cancer cells and sensitizing them to chemotherapeutic medicines [89,90,91,92,93,94,95,96]. For example, a study on *Daphne altaica* Pall, a traditional Kazhak medicine, has demonstrated the anticancer effect of the medicine in EC through modulating PPAR*γ*. The *D. altaica* extract (Da-Ea) has inhibited the cell proliferation of Eca-109 cells through upregulating PPAR*γ*, which also induced apoptosis and S phase cell cycle arrest [97]. According to another study, inhibiting FXR with FXR shRNA or guggulsterone reduced tumor cell survival and metastasis and induced apoptosis in vitro, as well as decreased EC growth in nude mice xenografts [64]. Therefore, we have also concluded the role of natural products in modulating the expression of NRs in EC. Additionally, we emphasize NRs’ potential to serve as a novel therapeutic target and their capacity to treat and prevent EC.

## 2. Nuclear Receptor Signaling

NRs are a class of TFs, which are significant in both the development and progression of cancer [50]. The majority of NRs activate transcription as either homodimers or heterodimers with the RXR, even though a small fraction of NRs can bind and stimulate transcription as monomers [42]. When the ligand is bound, NRs undergo a conformational shift that allows the recruitment of coactivator proteins, which stimulate transcription, and co-ordinately dissociate the corepressor [42]. NR family members control transcription in several ways and can both activate and repress gene expression [98]. A subset of NRs that heterodimerize with RXR, including LXR, RAR, and TR, can actively silence target genes by binding to HREs in the absence of ligands [99,100]. Other NRs, such as LXR, PPAR, and GR, can inhibit the actions of other TFs, such as activator protein-1 (AP-1) and nuclear factor (NF)-*κ*B, in a ligand-dependent manner [42].

**Figure 2:**
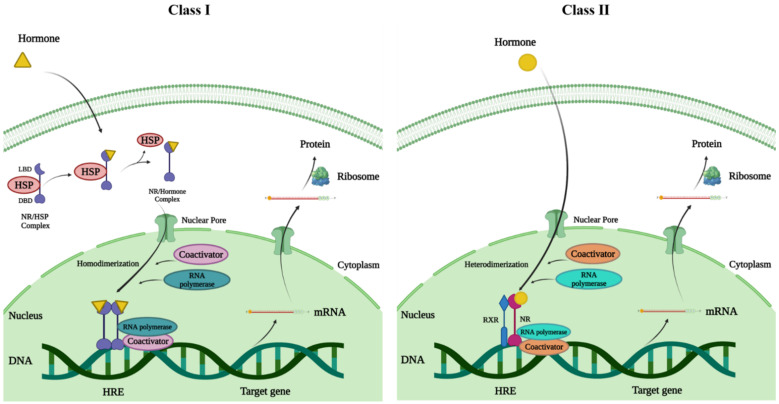
The mode of action of NRs; NRs exert their transcriptional stimulation of target genes via two distinct mechanisms. Class I—In the class I type, the ligand is bound in the cytoplasm, which causes the chaperons that are bound to the receptors to dissociate, causing the receptor to move and dimerize. Class II—For the class II type, the receptor dimerizes with another receptor (heterodimerization) to bind to nuclear response elements; ligand binding then releases the co-repressor, activating the receptor’s transcriptional unit.

NRs are divided into four categories based on their method of action: Type I-IV [101]. Type-I steroidal NRs, which include GR, PR, ER, and AR, are entrenched in the cytoplasmic membrane and connected to heat shock proteins (HSP90), and when the receptors bind to their ligand, the receptors release the chaperone, allowing homodimerization and are transported into the nucleus [101]. The type II category includes the non-steroidal NRs such as thyroid receptor, VDR, RAR, PPAR, and LXR, which are found in the nucleus and form an obligatory heterodimer with the RXR [102]. The heterodimeric complex is normally associated with corepressor proteins (e.g., NCoR and SMRT) in the absence of a ligand. However, when a ligand binds, corepressor proteins are released and coactivators are recruited, which modify chromatin structure and allow target genes to be activated [98,101,102] (Figure 2). The type III category contains orphan receptors which function as same as the Type I receptors but create a monomer that identifies an elusive DNA sequence, whereas Type IV, as monomers, attach to the half-site in HREs and function [38,101]. ER and AR signaling networks regulate reproduction, RAR and all-trans retinoic acid (ATRA) signaling pathways regulate mammalian embryonic development, VDR and metabolism regulate immune function and bone homeostasis, and GR and PPAR signaling pathways regulate inflammatory response [103,104,105,106,107,108,109,110]. Although ligands and coregulators are significant regulatory nodes in NR signaling pathways, different tissues and cell types have different ways of communicating the afferent physiologic signal through each channel [111].

## 3. Nuclear Receptors in Esophageal Cancer

**Figure 3:**
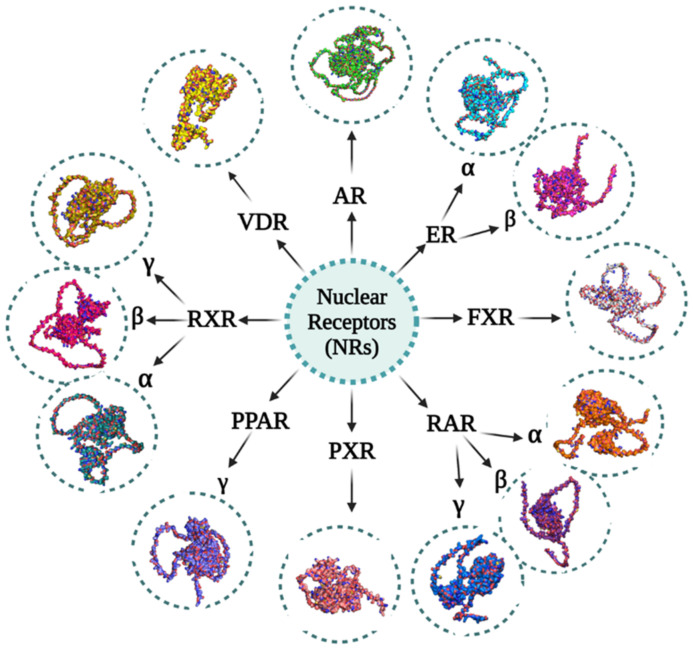
Various NRs involved in esophageal cancer and their 3D structures: Androgen receptors (ARs) (UniProt ID: P10275), Estrogen receptor alpha (ER*α*) (UniProt ID: P03372), Estrogen receptor beta (ER*β*) (UniProt ID: PQ92731), Farnesoid X receptors (FXRs) (UniProt ID: F1DAL1), Peroxisome proliferator-activated receptor gamma (PPAR*γ*) (UniProt ID: P37231), Pregnane X receptors (PXRs) (UniProt ID: F1DAL3), Retinoic acid receptor alpha (RAR*α*) (UniProt ID: P10276), Retinoic acid receptor beta (RAR*β*) (UniProt ID: P10826), Retinoic acid receptor gamma (RAR*γ*) (UniProt ID: P13631), Retinoid X receptor alpha (RXR*α*) (UniProt ID: P19793), Retinoid X receptor beta (RXR*β*) (UniProt ID: P28702), Retinoid X receptor gamma (RXR*γ*) (UniProt ID: P48443) and Vitamin D receptors (VDRs) (UniProt ID: P11473). These proteins’ primary structures were taken from the UniProt database. Using the AlphaFold protein structure database, the structures of these proteins were predicted. The image generation and visualization of the structures of these proteins were performed using PyMOL [47,48,115,116,117,118].

NRs are important not only in normal physiology but also in a variety of pathological disorders, the most prominent of which is cancer, where they regulate apoptosis, cellular growth, migration, and invasion [112]. NRs govern a range of biological activities that overlap with cancer cell characteristics; therefore, their functions in carcinogenesis and cancer progression have been extensively studied in recent decades [26,113]. Studies have shown that NRs expression and activation are highly expressed in cancer cells, and that leads to the survival of these cells. Therefore, inappropriate NR activation may contribute to the development and spread of cancer [114]. The significant role of numerous NRs such as ARs, ERs, FXRs, PPARs, RARs, RXRs, PXRs, and VDRs, has been identified in EC cells, and they likely contribute to the development and progression of this cancer by controlling several TFs and signaling pathways (Figure 3). In view of this, the main focus of this study is on relevant NRs associated with EC and the therapeutic value of utilizing small compounds such as agonists and antagonists. Table 1, Table 2 and Table 3 summarize the role of nuclear receptors in esophageal cancer in clinical, in vitro, and in vivo studies.

### 3.1. Androgen Receptors (ARs)

AR is a ligand-activated TFs in the steroid receptor family [180]. Growth factors, natural hormones, peptides, and synthetic compounds are all examples of ligands that can activate these receptors [180]. The AR is found in skeletal muscle, the prostate, the testes, the uterus, the breast, and other tissues [181]. The AR gene, which is 90 kb in size and situated on the X chromosome, is coded by eight exons [182]. Different domains in the AR include the N-terminal domain (NTD), DNA-binding domain (DBD), and ligand-binding domain (LBD). The least homologous section of the AR is its N-terminal region (amino acids 1–559), with less than 15–20% similarity among the class I members. AF-1, crucial for AR activity, is present in the NTD [183]. AR’s AF-1 contains all of AR’s phosphorylation sites except for three and is a target for several growth factors that phosphorylate the sites and activate the AR ligand on their own [184,185]. The DBD helps the AR to bind to the androgen Response Elements (ARE) in the regulatory regions of androgen-responsive genes. The DBD contains two zinc finger motifs necessary for DNA binding and dimerization and is highly conserved among receptors. The DBD and LBD’s lysine-rich hinge regions are essential for the nuclear localization of the receptor [186]. The AR’s LBD is responsible for ligand binding, is only minimally conserved among receptors, and contains AF-2, which is required for full receptor activation in the presence of ligand [180]. AF-2 refers to residues in the LBD that are implicated in transcription control. In a hormone-dependent way, this region of the AR recruits a set of coregulatory proteins known as p160 coactivators (e.g., steroid receptor coactivator-1 (SRC-1)) [187]. Interestingly, it was reported that AR is implicated in the development and progression of various cancer types, including prostate, breast, ovarian, etc. [188,189,190,191,192]. Therefore, AR’s expression and function are often investigated in cell lines and tumor specimens. However, the role of AR expression and function in the development and progression of EC is still poorly understood.

Increasing lines of evidence suggest that AR and AR responsive are highly overexpressed and activated in EC and controls the survival and prognosis of patients. For example, a study in 40 ESCC tumor tissues demonstrated high levels of AR expression in invasive ESCC tissues. In addition, this study also showed that the knockdown of the KYSE450 EC cell line with AR shRNA decreased the expression of AR, cell invasion, pAkt, and matrix metalloproteinase 2 (MMP2) [119]. Another clinical study showed high expression of AR in tissues from tobacco using ESCC patients compared with normal esophageal squamous tissues. Besides, higher expression of AR was also observed in the EC109, EC9706, HKESC-2, and TE-12 EC cell lines. Moreover, the inhibition of AR by shRNA reduced cell viability, cell growth, colony formation, anchorage-independent growth, and the S and G2/M phase. In addition, in mice with various androgen status, the overexpression of AR enhanced tumor growth. Further, AR promotes interleukin 6 (IL6), a common AR target gene in ESCC, transcription by binding directly to the IL6 promoter, and IL6 can then activate AR expression. Furthermore, prominent levels of AR and IL6 expression in human ESCC predict a worse clinical outcome in tobacco users [120]. Another clinical study demonstrated that AR gene expression was substantially higher in normal squamous epithelium than in esophageal adenocarcinomas [165]. According to another study, higher levels of dihydrotestosterone (DHT) inhibited the proliferation and cell division, induced cell cycle arrest and cell senescence and also altered androgen-responsive genes in OE33-AR, JH-AR, and OE19-AR EAC cell lines [148]. In addition, another study showed an increase of FK506-binding protein 5 (FKBP5), which is an androgen-responsive gene in AR-transduced OE33 cells (OE33-AR) [147]. Taken together, these findings demonstrated the significance of AR in the development and spread of EC, and additional research is required to identify the potential use AR as a therapeutic target in EAC and ESCC.

### 3.2. Estrogen Receptors (ERs)

Estrogen receptors (ERs) belong to the NR superfamily, which also comprises receptors that mediate the effects of thyroid hormones, steroid hormones, retinoids, and vitamin D [193]. ERs, similar to other steroid receptors, primarily serve as ligand-inducible TFs that bind chromatin at specific response regions as homodimers [193]. To interact with estrogen response elements (EREs) or other TFs, ERs dimerize and move to the nucleus, where they interact with them. This causes the recruitment of coregulatory proteins (coactivators or corepressors), an increase or decrease in mRNA levels and associated protein synthesis, as well as physiological responses [194,195,196]. The ligand-induced transcriptional activity of ER is mediated by two distinct activation functions, AF-1 and AF-2 [194,195,196]. ERs, similar to other members of the NR family, have structurally and functionally different domains. The DNA recognition and binding are carried out by the C or DNA-binding domain (DBD), which is the protein’s central and most conserved domain, while the COOH-terminal multifunctional D/E/F or ligand-binding domain (LBD) is responsible for ligand binding. The NH2-terminal or A/B domain is the least conserved and has the greatest variation in sequence and length [197,198]. Based on sequence homology with other receptors, the domains in the receptor have been split into six regions, A-F. Exon 1 codes for the N-terminal domain (regions A and B), exons 2 and 3 for the DNA-binding domain (region C), exon 4/hinge region (region D), and exons 5-8 for the hormone binding domain (regions E and F) [199]. ERs are divided into two subtypes: estrogen receptors *α* (ER*α*, also known as ER1 or ESR1) and estrogen receptors *β* (ER*β*, also known as ER2 or ESR2), which are encoded by the estrogen receptor 1 (ESR1) and 2 (ESR2) genes, respectively. They are members who belong to the NR superfamily and carry out a range of biological processes [194,200,201].

In humans, ER*α* and ER*β* play a critical role in the control of various intricate physiological processes. A multitude of disorders is linked to abnormal ER signaling, including cancer, metabolic and cardiovascular disease, neurodegeneration, inflammation, and osteoporosis [202,203,204]. For years, scientists have known that estrogen and its receptors play a critical role in cancer development [205]. Multiple investigations using esophageal tissues and various cell lines have demonstrated higher expression of both ER*α* and ER*β* at variable levels, pointing to the significance of ER in the development of EC. For example, a recent study on EC has proved that apart from typical risk factors, the hormonal environment may play a crucial role in EC development [206]. Studies have demonstrated that positive ER*α* expression in combination with negative ER*β* expression is an unfavorable independent prognostic predictor in ESCC [166,167]. In tumor tissues, the expression of ER*β* is higher in AC and poorly differentiated SCC, and it increases with tumor stage and dedifferentiation. As a result, ER*β* seems to be a sign of poor biological function, dedifferentiation, or a more advanced stage of disease [125]. Further studies on ESCC tissues showed that the levels of ER*α* and ER*β* were inversely connected, and the downregulation of ER*α* and the overexpression of ER*β* could indicate a poor prognosis [121]. Another study has demonstrated that the different isoforms of ER*β* (ER-B1, ER-B2, ER-B3, and ER-B5) were shown to be overexpressed in EA tissues and suggests a possible role of antiestrogens in the treatment of EA [124]. Interestingly, it was noted that in EC cells, estrogen ligands such as 17*β*-estradiol and selective estrogen receptor modulators (SERM) inhibited cell proliferation. The amount of anti-growth effects caused by receptor agonists was proportional to the quantity of ER expression in the cell lines. Therefore, this research revealed that selective ER ligand treatment in EC and BE cells results in decreased cell growth and induced apoptosis [126,150]. In a distinct study, 1, 3, 5-tris (4-hydroxyphenyl)-4-propyl-1H-pyrazole (PPT), an ER*α* agonist, was shown to reduce the number of ECGI10 + ER*α* cells. Moreover, estradiol significantly increased the cell proliferation of ECGI10 + ER*β* cells, and the addition of ICI 182780 dramatically reduced estradiol-mediated cell proliferation. In conclusion, this study’s findings unequivocally show that the presence of ER*β* was strongly correlated with poor prognosis in ESCC, possibly by affecting the proliferation of carcinoma cells [123]. Another study showed that the ER system contributes to the spread of EC, and a highly selective ER*α* antagonist (MPP) and an ER*β*-specific antagonist (PHTPP) elicited a concentration-dependent reduction in proliferation in EC cell lines. In addition, caspase 3/7 activity was significantly elevated in OE33 cell lines treated with MPP and PHTPP, and there was an increase in LDH activity in the presence of MPP-treated OE-33 cell lines [122]. However, a recent study showed that 17*β*-E2 inhibited the proliferation of human EC109 ESCC cells in a dose-dependent manner, which was inhibited by the ER antagonist ICI 182,780. Additionally, 17*β*-E2 significantly increased the release of intracellular Ca^2+^ and the entry of extracellular Ca^2+^ into ESCC cells, which was also inhibited by the ER antagonist IC1 82,780. When combined, this study shows that estrogen inhibits the proliferation of human ESCC cells, most likely via the ER-Ca^2+^ signaling pathway and it could a reason for the male predominance of ESCC [149]. In conclusion, it is evident that in a vast majority of cases of EC, ERs are markedly overexpressed and play a critical role in cell survival. Moreover, ES cancer cell invasion, migration, and proliferation have all been demonstrated to be inhibited by ER targeting, which also causes apoptosis. Additionally, the development of particular ER modulators would help in the prevention and treatment of ESCC patients.

### 3.3. Farnesoid X Receptors (FXRs)

The farnesoid X receptor (FXR) is a ligand-activated TF that belongs to the family of the NR, which is also classified as a nuclear bile acid (BA) receptor [207]. BAs operate as powerful endogenous ligands for FXR activation in the body [207]. FXR is a common receptor present in the intestine and liver that regulates bile acid, glucose, lipid metabolism, and energy balance to aid in maintaining systemic metabolic equilibrium [207,208]. FXR is encoded by the NR1H4 gene and controls the activities of several organs, including the brain, breast, cardiovascular system, gut, kidney, liver, and pancreas. As a result, FXR has become a popular therapeutic target for a wide range of disorders [207,208]. FXR detects physiologic and pathological metabolic changes and alters by regulating the transcription of genes related to cholesterol, fatty acid (FA), glucose, and amino acid balance. FXR*α* (NR1H4) and FXR*β* (NR1H5) are two FXR genes that have been discovered, and the FXR*α* gene encodes four physiologically active versions (FXR*α*1, *α*2, *α*3, *α*4) as a result of several promoters and RNA splicing [209,210]. FXR*α*1/*α*2 and FXR*α*3/*α*4 are expressed at equal levels in the liver, whereas FXR*α*3/*α*4 isoforms are mostly expressed in the gut [211]. FXR binds to DNA (i.e., FXR response elements) as a monomer or as a heterodimer with the retinoid X receptor (RXR), another ligand-activated TF [210]. The N-terminal ligand-independent transcriptional activation AF-1 domain, DBD, a hinge region, and the C-terminal LBD comprising a transcriptional AF-2 comprises the structure of FXR, which is the same as the typical NR structure [212]. The hinge region sequence and the length of the AF-1 region differ between the four FXR isoforms. FXR agonists bind to the pocket produced by LBD, promoting its binding to FXR response regions in downstream target genes, which stimulates transcriptional activation [213].

According to recent research, FXR overexpression has been linked to the development and progression of breast, lung, pancreas, and esophageal malignancies. It has also been linked to tissue and cell-specific involvement in a variety of malignancies. It was also noted that FXR is strongly expressed in esophagitis, BE, and EAC [39]. For example, a study has demonstrated that FXR is overexpressed in BE, and guggulsterone, an FXR antagonist, significantly enhances apoptosis in a human BE-derived cell line which implies that FXR may play a role in apoptosis regulation [127]. According to another similar study, the suppression of FXR with FXR shRNA or guggulsterone reduced tumor cell survival and metastasis and induced apoptosis in vitro, as well as decreased EC growth in nude mice xenografts [64]. Another study demonstrated that, FXR was expressed in GERD tissues, and the level of expression has greatly increased in esophagitis [128]. In addition, the same study showed that FXR and basal TLR2 expression were linked, and TLR2 and FXR were significantly elevated during reflux esophagitis [128]. On the contrary, an in vitro and in vivo investigation has reported that the activation of FXR performs an antitumor role in the ESCC. FXR activation by its ligand GW4064 inhibited the ERK1/2 pathway and cell growth, increased apoptosis, and caused cell cycle arrest in ESCC cells. Further, the FXR ligand GW4064 reduced the growth of ESCC in a mouse xenograft model [151]. Altogether, it was identified that FXR could be a potential target for the management of ESCC.

### 3.4. Peroxisome Proliferator-Activated Receptors (PPARs)

Peroxisome proliferator-activated receptors (PPARs) are fatty acid-activated TFs, which belong to the nuclear hormone receptor superfamily, that control energy metabolism. PPAR*α* (NR1C1), PPAR*γ* (NR2C2), and PPAR*δ* (NR3C3) (also known as PPAR*β*) are the three PPAR subtypes that have been discovered so far [214,215,216,217,218,219]. All PPARs, which have four functional domains termed A/B, C, D, and E/F, share the fundamental structural characteristics of the majority of NRs. The PPAR is phosphorylated by the ligand-independent AF-1 in the N-terminal (A/B) domain [220]. PPARs bind to the peroxisome proliferator response element (PPRE) in the promoter of PPAR target genes, and this interaction is mediated by the two-zinc fingered conserved core DBD, also referred to as the C domain. The cofactor docking site is the D domain, and the E domain is also known as the LBD. The E/F domain’s ligand-dependent AF-2 mediates the recruitment of PPAR cofactors involved in the transcription processes [220].

PPAR*α* and PPAR*δ* are also expressed in oxidative tissues and control gene expression involved in oxidative phosphorylation (OXPHOS), substrate delivery, and oxidation. PPAR*α* stimulates energy dissipation and is found mostly in the brown adipose tissue (BAT), gut, heart, kidney, liver, and skeletal muscles [221,222]. PPAR*α* influences esterification, fatty acid transport, and oxidation to mediate its actions. PPAR*β*/*δ* is widely expressed and plays a role in fatty acid oxidation as well as blood glucose control. White adipose tissue (WAT) has the highest levels of PPAR*γ* expression, which is largely engaged in energy storage through promoting adipogenesis and lipid synthesis [220]. The PPAR*γ* is expressed mainly in the gut, immune cells, liver, and skeletal muscles [221,223].

The binding of cognate lipid ligands, heterodimerization with another NR (RXR), the interaction of a few transcriptional coactivators, including PPAR coactivator-1 (PGC-1), as well as binding of the complex to PPAR response elements (PPREs) in the promoter of target genes are necessary for PPARs to function as NRs for transcription [223]. PPARs are triggered by several ligands. Eicosanoids and long-chain fatty acids (FAs) are examples of some common endogenous ligands for PPAR*α* and PPAR*β*/*δ*, PPAR*γ* on the other hand, is activated by arachidonic acid metabolites [224,225]. Pioglitazone, GW1929, and GW2090 are anti-diabetic thiazolidinedione (TZD) substances that specifically activate PPAR*γ*, whereas GW501516 is a highly selective PPAR*β*/*δ* ligand [216,226].

The activation of PPAR by ligands has been linked with several malignancies. In vitro investigations on human cancer cells indicated growth-inhibitory effects such as cell-cycle arrest, differentiation, and death induced by PPAR ligands [227]. For example, a study has demonstrated the expression of PPAR*γ* in T. Tn, and EC-GI-10 ESCC cell lines and revealed the marked growth inhibitory ability of PPAR*γ*-ligands (Troglitazone, Pioglitazone, and 15d-PGJ2) to prevent the growth of human ESCC. Moreover, this effect was evident by the dose-dependent inhibition of deoxyribonucleic acid synthesis and G1 arrest and an increased level of cyclin-dependent kinase inhibitor p27 (Kip1), p21 (Cip1/Waf1), and p18(Ink4c). In addition, troglitazone treatment increased the expression of interleukin-1 alpha in EC-G1-10 cells [152]. Similarly, another study showed that troglitazone, a PPAR*γ*-ligand, treatment in TE-13 cells inhibited the development of human ESCC through G1 cell cycle arrest by increasing p27 expression and induced apoptosis by increasing the expression of Bid, Bax, PARP, and caspase 3 and reducing the expression of cyclin E, MDM2, p16, cytochrome C, caspase 8, and Bcl-XL [153]. Interestingly, another study using 55 primary ESCC tissue samples has shown that the expression level of PPAR*γ* mRNA was decreased in ESCC compared with normal esophageal mucosa, and this was correlated with poor prognosis [129]. Moreover, PPAR*γ* and SIRT1 were substantially expressed in ESCC tissues, but high PPAR*γ* expression was correlated with tumor grading but not with poor prognosis [168]. In this study, it was observed that increased tumor growth and poor prognosis were associated with the high expression of SIRT1, a protein that supports cell survival and angiogenesis in ESCC patients. However, SIRT1 expression was positively linked with EGFR but not with PPAR*γ* or survivin [168]. In another study, it was observed that miR-10b was elevated while the expression of PPAR*γ* was downregulated in EC tissues and ESCC cell lines EC109 and TE10, which established that PPAR*γ* is a legitimate miR-10b target. Additionally, miR-10b suppression improved the chemosensitivity of EC cells to DDP in vitro and in vivo, and the overexpression of miR-10b decreased the PPAR*γ*-mediated DDP sensitivity. The Akt/mTOR/p70S6K signaling pathway was also activated as a result of the overexpression of miR10b, and the deactivation of Akt/mTOR/p70S6K by Akt inhibitor (GSK690693) reduced miR-10b-induced DDP resistance in EC cells. Together, these findings show that PPAR*γ* inhibition by miR-10b increased DDP resistance in EC by increasing Akt/mTOR/P70S6K signaling. Moreover, it was observed that after DDP treatment, the activation of PPAR*γ* significantly aided DDP-induced apoptosis in EC109 and TE10 cells. In addition, elevated PPAR*γ* consistently resulted in a rise in Bax levels and a decrease in Bcl2 levels after DDP treatment [133]. Interestingly, lycopene, a natural compound, was shown to suppress NF-*κ*B and COX-2 expression and enhance the protein expression of PPAR*γ* and cleaved caspase 3, which leads to an increase in apoptotic proteins and a decrease in inflammatory cytokines. These findings showed that an effective amount of lycopene could prevent the development of EC in NMBzA-injected F344 rats through potential anti-inflammatory and pro-apoptotic pathways [155]. Another study showed that Da Ea (ethyl acetate extract of *D. altaica*), which has anti-cancer effects, increased PPAR*γ* expression levels, induced apoptosis and S phase cell cycle arrest, which prevented the proliferation of ECA 109 cells [97]. In addition, an in vitro and in vivo study in EC cells and ESO26 cells injected mice treated with T0070907 has demonstrated the transcriptional feedback loop between the PPAR*γ* and the master regulator transcription factors (MRTF) that are particular to EC and fatty acid production. PPAR*γ* overexpression was caused by MRTFs functioning together to promote PPAR*γ* transcription by directly controlling its promoter and a distal EAC-specific enhancer. Moreover, this study also shows a decrease in cell proliferation and induced apoptosis in T0070907 treated OE33 AND ESO26 cell lines. In addition, in vivo study has demonstrated a decrease in the expression of FASN, ACC, ACLY, SCD and tumor growth in ESO26 cells injected mice [156]. Another study showed increased expressions of PPAR*γ,* COX-2, HGF, gastrin, and NF-*κ*B activity in BE tissues. Moreover, the increased NF-*κ*B activity is probably linked to increased IL-8 and COX-2 expression [130]. Similarly, in EC tissues, upregulation of PPAR*γ* was observed, and the treatment of EC cell lines with PPAR*γ* antagonists (T0070907 and GW9662) decreased EC cell adhesion, expression of p-focal adhesion kinase (p-FAK) and pERK and induced apoptosis [131]. Another study has reported the reduced expression of PPAR*γ* in esophageal tumor lesions and proved that ESCC cell proliferation could be inhibited by efatutazone, a PPAR*γ* agonist, by inactivating the PI3K–Akt and MAPK pathways [154]. Interestingly, an in vitro and in vivo study demonstrated that the activation of PPAR*γ* inhibits cancer cell growth in vitro by inducing apoptosis through increasing caspase 3 activity, but systemic PPAR*γ* activation increased the growth of OE33-derived transplantable adenocarcinomas in vivo due to increased cell proliferation [132]. Collectively, these data suggest that PPARs play a critical role in the emergence of EC and might serve as a novel therapeutic target.

### 3.5. Retinoic Acid Receptors (RARs)

RARs are TFs that belong to the NR superfamily which can have non-genomic effects by triggering kinase signaling pathways that regulate the transcription of RA target genes [228,229]. RARs have a significant role in a variety of physiological processes, including embryonic development and organ homeostasis. RARs also help to regulate gene networks that control cell growth, differentiation, survival, and cell death at the cellular level [228,229]. RARs are divided into three different subtypes: RAR*α*, RAR*β*, and RAR*γ* and each subtype has different isoforms. RAR*β* is divided into four isoforms (*β*1, *β*2, *β*3, and *β*4), each with differing affinities for retinoids and biological roles [230]. The first nuclear RAR in humans, RAR*α* (NR1B1), has a high affinity for ATRA and has preserved the NR modular organization structure. RAR*β* (NR1B2) and the RAR*γ* (NR1B3) are the second and the third RAR gene respectively [228].

RAR’s modular structure, which includes many domains and functions, allows them to process both ligand binding and transcription [231]. The transactivation domain, AF-1, is found in the amino terminus (A/B region) and forms a recognition surface for co-activators and other TFs [231]. For DNA recognition, the DBD holds two zinc finger motifs, and the LBD of the family members are highly conserved. It has a ligand-induced activation factor called AF-2, which is important in transcriptional coregulator interactions [231]. RARs can bind to specific enhancer regions in DNA, known as retinoic acid response elements (RAREs) in target gene promoters, after dimerization with RXR, resulting in transcriptional activation of target genes in the presence of ligand [228,232].

Retinoids can induce cell differentiation and inhibit proliferation, which is one of the reasons why they are used to treat cancer [233]. Surfeit numbers of clinical evidence have demonstrated that RAR*β*2 expression is usually inversely linked with tumor grade and frequently lost or epigenetically silenced in human malignancies [230,234]. According to a clinical investigation, the state of squamous differentiation and the increase in RAR*β*-expression are early events connected to EC [169]. Several clinical, in vitro*,* and in vivo studies have reported the leading role of RARs in the development and growth of EC cells. For example, it was found that expression levels of RAR*α* and RAR*β* increased significantly in the higher stages of Barrett’s adenocarcinoma while expression of RAR*γ* was significantly reduced. Therefore, RAR*γ* may have a tumor suppressor role in Barrett’s carcinogenesis [135]. In EC cases, RAR*β*2 mRNA expressions were markedly decreased, whereas RAR*β*4 mRNA expression was elevated. Additionally, when compared to normal tissues, tumors had higher expressions of cyclin D1 and EGFR, while lower expressions of RAR*β*1, COUP-TFI (COUP transcription factor 1), and COUP-TFII were observed. Therefore, in tumor samples, decreased RAR*β*2 expression was linked with increased RAR*β*4 expression and the inhibition of COUP-TFI and COUP-TFII [141]. Another study has proven that RAR*α* was overexpressed in human EC tissues, and further, it was demonstrated that RAR*α* knockdown by siRNA inhibited EC cell proliferation by downregulating proliferating cell nuclear antigen (PCNA), Ki67, MMP7, and MMP9 expression and increased the drug sensitivity to 5-fluorouracil and cisplatin [136]. Benzo-[a]pyrene diol epoxide (BPDE) is found to be an active metabolite of tobacco procarcinogens, and a study has proven that by suppressing RAR*β2* transcription, BPDE reduced RAR*β2* mRNA and protein levels. Moreover, retinoic acid was able to partially block BPDE’s inhibitory effect on RAR*β*2 expression while increasing the cell cycle G1 phase. Additionally, BPDE-induced COX-2 expression was linked to RAR*β*2 inhibition. The expression of EGFR, ERK1/2 phosphorylation, c-Jun, and COX-2 were decreased after the RAR*β*2-expression vector was transfected into EC cells. Additionally, there was little change in the expression of c-Jun and COX-2 after co-treatment of RAR*β*2 positive cells with BPDE. These studies have proved that BPDE may cause EC via inhibiting RAR*β*2 [158,161,163]. Another study showed that RAR*β2′*s tumor suppressor function may be linked to its ability to decrease COX-2 expression, which plays a role in carcinogenesis and metastasis, and 13 *cis*-RA mediated activation of RAR*β2* suppressed COX-2 expression, implying that COX-2 inhibition is dependent on RAR*β2* expression. BPDE significantly caused time-dependent methylation of the RAR*β*2 gene promoter in esophageal cancer cells, as well as suppression of EGFR, ERK1/2 phosphorylation, c-Jun, and COX-2 expression. RAR*β*2 expression is decreased by BPDE, and the restoration of RAR*β*2 expression lowers COX-2 protein in esophageal cancer cells, implying that RAR*β*2 plays a significant role in preventing esophageal carcinogenesis [159,162]. It was also observed that RAR*β* expression was gradually lost, starting with the mildly dysplastic stage of esophageal mucosae. Additionally, the expression of RAR*β* was reduced as a result of the differentiation of esophageal squamous. Further, P53 and Ki67 were accumulated in the later precancerous stage of EC. This study suggests that the expression of RAR*β*, P53, and Ki67 could be used as biomarkers for early EC diagnosis in high-risk populations [137,170,172]. In ESCC, DNA methylation frequently results in the inactivation of the genes RAR*β*, RAR*β*2, CRBP1, and TIG1, which are linked to retinoic acid signaling, and in contrast, another study revealed that RAR*β*2, p16, MGMT, CLDN3, CRBP, and MT1G were increased in ESCC tissues [171,173]. In mice tumors, 4- nitroquinoline 1-oxide (4-NQO), a carcinogen, inhibited RAR*β*2 but increased the expression of p-ERK1/2, c-FOS, and COX-2 proteins, as well as the methylation of the RAR*β*2 gene promoter. Moreover, it was shown that RAR*β*2 expression was decreased and p-ERK1/2, and COX-2 expression were increased by treatment with 4-NQO in human EC cells in vitro. Moreover, upregulated p-ERK1/2 and COX-2 expression were found in EC tissues, and p-ERK1/2 expressions were linked to a more advanced clinical tumor stage [164]. In addition, it was observed that overexpression of RAR*β*2 induced retinoid receptor-induced gene 1 (RRIG1) and inhibited Erk1/2 phosphorylation and COX-2 expression [142]. Another study showed that the knockdown of DNA (cytosine-5)-methyltransferase1 (DNMT1) in KYSE30 and TE-1 EC cells led to promoter demethylation and RAR*β* overexpression. This study showed that smoking status and low RAR*β* expression were associated with DNMT1 overexpression in esophageal SCC patients. Through the activation of DNMT1 in esophageal squamous epithelial cells, NNK, a tobacco-specific carcinogen, might cause RAR*β* promoter hypermethylation, which ultimately increased cell proliferation and inhibited apoptosis [138]. Another study showed that *N*-(4-hydroxyphenyl) retinamide (4HPR) but not RA suppressed the proliferation of the ESCC cell line EC109 in vitro. In addition, RAR*β*2 induction is correlated with growth inhibition in RA-responsive cells, whereas a failure in RAR*β*2 inducibility is correlated with RA resistance. These results suggest that 4HPR may operate as a growth inhibitor through direct or indirect interactions with RAR*β*2 [160]. Several clinical studies investigated the methylation status and the expression of the RAR*β*2 promoter area and revealed a significant relationship between RAR*β*2 methylation status and tumor grade. Further, only G2 stage (intermediate grade) tumors showed a link between methylation status and lower expression of RAR*β*2, and its restoration was accompanied by growth inhibition after 5-aza-dc treatment [139,140,174]. Therefore, RARs (*α*, *β*, *γ*) can be targeted and used as markers for the prevention and treatment of both EACs and ESCCs.

### 3.6. Retinoid X Receptors (RXRs)

Retinoid X receptors (RXRs) are heterodimeric partners of other members of the NR superfamily [235]. There are three types of RXRs: RXR*α*, RXR*β,* and RXR*γ*, all of which are nuclear transcriptional transactivator proteins that bind to DNA and are ligand-dependent [175]. The “permissive” subclass of heterodimers, such as PPAR, LXR, and FXR, is transcriptionally activated by RXR ligands (“rexinoids”) either independently or in conjunction with partner ligands in the “non-permissive” subclass, such as RAR, VDR, and TR [235]. The morphogenesis, development, growth, and differentiation of cells are all regulated by RXR, and its expression is found to be altered in several solid tumors [175]. RXR modulators have therapeutic potential for cancer and other disorders involving the acquisition and disposal of nutrients, such as metabolic diseases [236].

A surfeit number of studies have proven that RXR is essential for the development of EC. For example, in a study, it was demonstrated that the mRNA expression of the three different subtypes of RXR is significantly different in EC tissues and RXR mRNA expression levels may be useful biomarkers for BE and related adenocarcinoma since changes in the mRNA expression of all three RXR subtypes (RXR*α*, RXR*β*, and RXR*γ*) are frequently observed in the development and progression of these diseases [175]. According to another study, EC tissues had higher levels of RXR mRNA and protein than normal esophageal tissues. The level of RXR overexpression was linked to tumor differentiation, TNM stage, and lymph node metastasis in EC patients. Further, EC patients with high RXR expression had considerably worse disease-free survival (DFS) and overall survival rates (OS). Moreover, multivariate analysis showed that the expression of RXR may be a predictor of DFS and OS in EC patients [176]. Another study showed that all six retinoid receptor subtypes, including RXR, were active in the tissues of EC patients. RXR*β* was inversely correlated with patient lymph node metastatic status and was linked with a better clinical outcome across these receptor subtypes. According to these findings, retinoid receptors, particularly, RXRs play significant roles in ESCC and are associated with patient prognosis [172]. Furthermore, another study has revealed that both mRNA and protein of PPAR*γ* and RXR*α* were expressed in ESCC cell lines from the KYSE series. Moreover, EC cell growth was decreased by the PPAR*γ* ligand troglitazone (TRO), and RXR*α* ligand 9-cis retinoic acid (9CRA) administration had a synergistic impact. The combined treatment with TRO and 9CRA, which also markedly elevated the sub-G1 phase, showed that ligand administration was predominantly responsible for inducing apoptotic cell death in EC cells. Additionally, TRO + 9CRA treatment significantly inhibited the growth of tumors implanted in nude mice [157].

### 3.7. Vitamin D Receptor (VDR)

The vitamin D receptor (VDR) belongs to the NR superfamily and is involved in vitamin D’s biological activities [237]. The VDR ligand regulates the expression of many genes involved in calcium/phosphate balance, cellular proliferation and differentiation, and immunological response [237]. VDR is abundantly expressed in cardiomyocytes, vascular endothelial cells, and vascular smooth muscle cells [238]. One of three retinoid X receptors (RXR*α*, RXR*β*, and RXR*γ*) forms dimers with VDR. The VDR homodimer or VDR-RXR heterodimer attaches to vitamin D response elements (VDREs), which are enhancer elements [239]. In combination with the RXR, ligand binding induces VDR nuclear localization and promotes VDR–DNA complexation. Particular VDREs have been discovered in the promoter sequences of genes that are activated or repressed by VDR. Interactions with coregulators are required for VDR-mediated gene regulation (coactivator and corepressor) [240]. The natural ligand of the VDR is 1,25-dihydroxy vitamin D (1,25(OH)_2_D_3_), a hormonal metabolite of vitamin D. VDR enters the nucleus after binding to 1,25(OH)_2_D_3_ and forms a heterodimer with retinoid X receptor (RXR), which regulates gene transcription by interacting with response elements in target gene promoters [241].

An N-terminal domain, a conserved DNA-binding domain, a flexible hinge region, and a conserved ligand-binding domain make up VDR’s structure [241,242]. The LBD has 12 helices and takes the form of a small, 3D structure when bound to a ligand. Deep inside the receptor, the ligand-binding pocket enables highly selective interactions with natural ligands such as 1,25(OH)_2_D_3_ [240].

In a study, it was demonstrated that through a bile acid ligand, VDR plays a role in the early development of EC. Interestingly, it has been shown that in both EAC and columnar cell metaplasia (CCM), VDR expression was considerably higher in male patients than in females. Moreover, VDR amplification was linked to a worse prognosis but not VDR protein expression [144]. However, another study has shown that both JNK1 and VDR were decreased in ESCC epithelial cells in comparison to the normal esophagus. JNK1 and VDR stromal expression also reduced the motility, migration, and proliferation of ESCC cells by blocking signaling pathways involved in proliferation and metastasis. Therefore, stromal JNK1 and VDR function as tumor suppressors in ESCC, and the degree of their stromal expression may affect the prognosis of ESCC [146]. Moreover, it was observed that EAC exhibits VDR expression, and as the tumor dedifferentiates, the expression level of VDR also decreases [143,177]. In contrast, another clinical study has demonstrated that the mRNA expression of VDR was higher in BE tissues compared to the normal squamous epithelium tissues [145]. In addition, it was shown that variable polymorphisms in genes involved in vitamin D metabolism are connected to the probability of reflux-BE-EAC development. In addition, low expression of VDR and CYP27B1 and high expression of CYP24A1 were observed in EAC tumor tissues compared to normal esophageal tissues [179]. Another study showed that claudin-2 was found to be strongly expressed in EAC and ESCC tissues, and its expression was linked to the expression of the bile acid receptors VDR and TGR5 [178]. These studies showed that dysregulation of VDR plays a critical role in the development of EC.

### 3.8. Other Nuclear Receptors

Several other NRs have also been thoroughly investigated and examined for their crucial function in esophageal carcinogenesis. One such receptor is the pregnane X receptor (PXR, NR1I2), also known as PAR (the receptor activated by pregnane) and SXR (steroid and xenobiotic receptor), which is the NR super family’s archetypal member [243]. Both endobiotics and xenobiotics can activate PXR. PXR’s biological function as a major xenobiotic receptor is primarily mediated by its ligand-dependent binding to regulatory gene sequences [244]. The 50 kDa PXR protein is composed of the DBD, the relatively short hinge region, and LBD with AF-1 and AF-2 regions [245]. PXR signaling has also been linked to cancer-related processes such as cell survival, proliferation, angiogenesis, and oxidative stress [246]. It was noted that PXR is associated with the development of EC. For example, a study has reported that PXR is highly overexpressed in BE and EAC patients and revealed their nuclear localization in adenocarcinoma tissues. Furthermore, PXR translocates to the nuclei of adenocarcinoma cells after being stimulated with lithocholic acid. This result, together with the discovery of a link between a PXR polymorphism and BE, suggests that PXR may have a role in esophageal illness prognosis and treatment [134]. Hence, insights into NRs and how they interact with TFs can lead to the discovery of novel drug targets that can be used to treat esophageal carcinogenesis.

## 4. NRs as Biomarkers in Esophageal Cancer

Biomarkers are any objective testing and evaluation characteristics that serve as indications for normal biological processes, case processes, or pharmaceutical responses [247]. Biomarkers are widely used for human illness investigation and play an essential role in early diagnosis, disease prevention, and the discovery of targeted drugs and drug reactions [247]. Patients with poorly differentiated esophageal carcinoma often have a bad prognosis, and various agonists and antagonists of NRs have been widely used for the treatment of EC. Agonists and antagonists with the structure-based design that can either induce or impede NR activity will give practical treatment methods for this disease [248]. The role of NRs as a potential biomarker has been suggested by the research undertaken for the early detection, prevention, and treatment of esophageal carcinogenesis. Moreover, some studies noted that AR is implicated in tumor growth, so it could be a good target for molecularly targeted ESCC therapies [119]. For example, a study on 77 EAC patients reported that 94.7% (75/77) of them were seen with high AR expression, and it also implies that AR influences overall survival. Therefore, this study suggests new treatment options for EAC, such as drugs that target AR signaling or androgen-responsive genes [147]. In tumor tissues, the ER*β* is expressed more in AC and in poorly differentiated SCC. ER seems to be a marker of poor biological behaviour, such as dedifferentiation or an advanced stage of disease ER expression rises with tumor stage and dedifferentiation in both AC and SCC [125]. In EC tissues, the low expression of PPAR*γ* was observed compared with normal esophageal epithelium; therefore, PPAR*γ* mRNA expression level can act as a prognostic marker in post-operative EC patients [129]. According to another study, Barrett’s tissues have significantly different levels of RAR mRNA expression than normal esophageal tissues, while Barrett’s dysplasia and adenocarcinoma tissues have dramatically different RAR mRNA levels. Therefore, these findings suggest that RAR mRNA levels may be useful biomarkers for this disease [135]. Even though NRs have the potential to serve as biomarkers and have been the subject of numerous studies, further clinical research is required to demonstrate their ability to serve as diagnostic and prognostic biomarkers for the treatment of EC.

## 5. Epigenetic Alterations in NRs in Esophageal Cancer

The majority of epigenetic regulation of gene expression is dependent on DNA methylation and histone modifications because there are no inherent changes in the DNA sequence [249,250,251]. In human tumors, abnormal epigenetic alterations are more common than gene mutations, and in the preliminary stages of cancer, epigenetic dysregulation is a common event [252,253,254]. Therefore, the disruption of the “epigenetic machinery” is significant in the genesis of cancer [249,255]. It is well-established that epigenetic changes play a crucial role in the development and progression of EC [256]. Further, epigenetic changes, especially in the form of DNA hypermethylation of tumor suppressor genes, have been seen in ESCC and EAC, as well as the EAC precursor lesion BE. A subset of these abnormal methylation of tumor suppressor genes is thought to be involved in the etiology of esophageal malignancies [257,258]. In a study, 125 ESCC tissues were analyzed and found that 98/125 patients (78.4%) had RAR*β*2 hypermethylation and concluded that hypermethylation of the tumor suppressor gene RAR*β*2 has been linked to the onset and severity of ESCC [174]. Another similar study reported that RAR*β*2 gene methylation is frequent in the esophageal mucosa of ESCC patients, and it tends to grow in prevalence in mucosal foci as the disease’s histological severity worsens [173]. In another investigation of 28 ESCC tissues, diminished RAR*β* expression was found in 42.9% (12/28) of the cases, with half of these instances showing RAR*β* DNA methylation. Although RAR*β* DNA methylation was found in non-neoplastic samples (10.0%), the incidence of methylation in ESCC (25.0%) was higher, suggesting that methylation of the RAR*β* gene may play a pivotal role in esophageal carcinogenesis [171].

## 6. Conclusions

ECs are one of the most aggressive and poorly understood deadliest diseases worldwide and better treatment options are urgently needed. Despite the recent advances in oncological and surgical treatment, new methods for predicting disease outbreaks and curing EC must be investigated to enhance results. For developing a better treatment system, understanding the vital roles played by the proteins and genes that are being altered due to epigenetics and mutations is necessary. NRs play a key role in the initiation, development, and progression of various cancers. Some of the NRs involved in EC development are ARs, ERs, FXRs, PPARs, RARs, RXRs, and VDRs, and their subtypes. A surfeit number of studies have reported the role of these receptors in esophageal carcinogenesis, and are expressed differently depending on the receptor’s function. Interestingly, a few studies have shown that NRs exhibit both tumor-promoting and tumor-inhibiting properties. In most cases, the majority of the NRs (ARs, ERs, PPARs, and RARs) are overexpressed in the ESCC and EAC tissues and cell lines. Moreover, NRs are also involved in the development, growth, and progression of ESCC and EA. This implies that NRs can be a suitable target for the diagnosis and prognosis of EC. Further, NRs also has the potential to be a biomarker for the early detection, prevention, and treatment of esophageal carcinogenesis. Multiple studies have reported the importance of agonists and antagonists that play a vital role in the function and expression of NRs in EC. Agonists and antagonists have the potential to inhibit tumor growth, migration, and invasion and cause apoptosis by modifying the expression of NRs and by controlling a wide range of genes involved in cell differentiation, proliferation, and apoptosis. Furthermore, epigenetic changes also play a significant role in the development and progression of EC. Several studies have proved that hypermethylation alters the expression patterns of RAR*β* and RAR*β*2 in EC [171,173,174]. It is also noted that there are very few major clinical trials conducted on NRs’ influence on EC. Therefore, more clinical studies on the role of NRs in the development of EC will aid in the discovery of novel therapeutic targets for better management of this disease.

## Figures and Tables

**Table 1:** Nuclear receptor (NR) expression in esophageal cancer and various ESCC and EAC cell lines.

Nuclear Receptors (NRs)	In Vitro*/*In Vivo*/* Clinical	Model/Cell Lines/Tissues	Expression (Up/Downregulation)	References
AR	Clinical	ESCC tissues	Up	[119]
	Clinical	ESCC tissues	Up	[120]
	In vitro	EC109, EC9706, HKESC-2, TE12	Up	[120]
ER*α*	Clinical	ESCC tissues	Down	[121]
	Clinical	EC tissues	Up	[122]
	In vitro	EC-GI-10 + ER*α*	Up	[123]
ER*β*	Clinical	EAC tissues	Up	[124]
	Clinical	EAC tissues	Up	[125]
	Clinical	ESCC tissues	Up	[121]
	Clinical	EC tissues	Up	[122]
	In vitro	EC-GI-10 + ER*β*	Up	[123]
	Clinical	ESCC tissues	Up	[126]
FXR	Clinical	BE and EAC tissues	Up	[127]
	Clinical	EAC tissues	Up	[64]
	Clinical	GERD tissues	Up	[128]
PPAR*γ*	Clinical	ESCC tissues	Down	[129]
	Clinical	BE tissues	Up	[130]
	Clinical	EC tissues	Up	[131]
	Clinical	BE tissues	Up	[132]
	Clinical	EC tissues	Down	[133]
PXR	Clinical	EAC tissues	Up	[134]
RAR*α*	Clinical	EC tissues	Up	[135]
	Clinical	EC tissues	Up	[136]
RAR*β*	Clinical	EC tissue	Up	[135]
	Clinical	EC tissues	Down	[137]
	Clinical	ESCC tissues	Down	[138]
RAR*β*2	Clinical	ESCC tissues	Down	[139]
	In vitro	KYSE410, KYSE510, COLO680N	Down	[140]
	Clinical	ESCC tissues	Down	[141]
	Clinical	ESCC tissues	Up	[142]
RAR*β*4	Clinical	ESCC tissues	Up	[141]
RAR*γ*	Clinical	EC tissues	Down	[135]
VDR	Clinical	EAC tissues	Down	[143]
	Clinical	EAC tissues	Up	[144]
	Clinical	BE tissues	Up	[145]
	Clinical	ESCC tissues	Down	[146]

**Table 2:** Mechanistic role of various nuclear receptors (NRs) in esophageal cancer in the presence of their agonists/antagonists.

Nuclear Receptors (NRs)	In Vitro/ In Vivo	Model/Cell Lines	Agonists/Antagonists	Results	References
AR	In vitro	OE33-AR	DHT	↑ FKBP5, HMOX1, FBXO32, WNT5A, VEGFA, KLK3	[147]
	In vitro	KYSE450	siRNA duplexes	↓ AR, Cell invasion, MMP2, p-Akt	[119]
	In vitro	EC9706	shRNA	↓ AR, Cell viability, Cell growth, Colony formation, Anchorage-independent growth, S and G2/M phase, IL-6, TNF ↑ G1/G0 arrest	[120]
		TE-1	pPYCAGIP-AR-GFP	↑ AR, Anchorage-independent growth, Cell growth, Colony formation, S and G2/M phase, IL-6 ↓ G1/G0 arrest	
		EC9706 and HKESC-2	siRNA	↓ AR	
	In vivo	EC9706-shAR cells injected mice TE-1 cells injected mice		↓ Tumor size and weight ↑ Tumor size and weight	[120]
	In vitro	OE33-AR, JH-AR, OE19-AR	DHT	↓ Cell proliferation, NDRG1 ↑ FKBP5, HMOX1, Cell Cycle Arrest, Cell Senescence	[148]
		OE33-AR	Enzalutamide	↑ Cell count ↓ FKBP5	
ER	In vitro	EC109	17*β*-estradiol	↓ Cell proliferation	[149]
				↑ Ca^2+^ signaling	
	In vitro	OE-19 and OE-33	17*β*-estradiol/SERM	↓ Cell growth ↑Apoptosis	[150]
			Tamoxifen	↓ Cell growth	
			Raloxifene	↓ Cell number, Ki67 ↑ E-cadherin, Apoptosis	
ER*α*	In vitro	EC-GI-10+ER*α*	Propyl-pyrazole-triol	↓ Cell proliferation	[123]
	In vitro	OE-19, OE-33	MPP	↓ Cell proliferation	[122]
		OE-33	MPP + E2	↑ Apoptosis, Caspase 3/7, LDH activity	
ER*β*	In vitro	EC-GI-10+ER*β*	Estradiol, DPN	↑ Cell proliferation	[123]
			ICI1 82,780	↓ Cell proliferation	
	In vitro	OE-19, OE-33	PHTPP	↓ Cell proliferation	[122]
		OE-33	PHTPP + E2	↑ Apoptosis, Caspase 3/7	
FXR	In vitro	TE-3, TE-12, SKGT-5	Guggulsterone	↓ FXR, Cell viability, COX-2, MMP-9	[64]
				↑ Apoptosis	
			Chenodeoxycholic acid	↑ FXR, COX-2 ↓ RAR-*β*2	
		SKGT-4 cells	sh-RNA	↓ FXR, Cell growth	
	In vivo	SKGT-4 cells injected mice	FXR shRNA/ Guggulsterone	↓ Tumor formation, Tumor growth	[64]
	In vitro	KYSE150, EC109, TE-1	GW4064	↓ Cell proliferation, Migration, pERK1/2 ↑ G0/G1 arrest	[151]
		KYSE150	GW4064	↑ p53, Caspase 3, Cleaved-PARP, SHP, BSEP ↓ c-fos, CyclinD1, IL-6, MMP7, TNF-*α*-induced proinflammatory genes levels	
		EC109	GW4064	↑ p21, p53, Bak1, Bim, Bax, Caspase 3, Cleaved-PARP ↓ IP-10, TNF-*α* levels	
	In vivo	BALB-C nude mice (EC109 xenografts)	GW4064	↓ Tumor volume, Tumor weight, pERK1/2	[151]
PPAR*γ*	In vitro	T. Tn	Troglitazone	↑ p27^Kip1^, p21Cip1^/Waf1^, p18Ink4c, G1 arrest ↓ DNA synthesis	[152]
		EC-GI-10	Troglitazone	↑ IL-1*α*	
		T. Tn	Pioglitazone, 15d-PGJ2	↑ Cell cycle arrest in G1 phase	
	In vitro	TE-1, TE-7, TE-8, TE-12, TE-13	Troglitazone	↓ Cell growth, Cyclin E, p16, MDM2, Cyt C, Caspase 8, Bcl-XL	[153]
				↑ p27, G1 arrest, Bid, Bax, PARP, Caspase 3	
	In vitro	OE33cells	Ciglitazone	↓ Cell proliferation	[130]
				↑ Caspase 3	
	In vitro	KYSE70	T0070907 and GW9662	↓ Cell adherence, pERK, pFAK ↑Morphological changes, Apoptosis	[131]
	In vitro	OE33	Pioglitazone	↑ PPAR*γ*, Apoptosis, Caspase 3 activity	[132]
				↓ Cell growth	
	In vivo	OE33 cells injected nude mice	Pioglitazone	↑ Tumor development ↓ Apoptosis, Insulin level	[132]
	In vitro	TE series	Efatutazone, sh-RNA	↓ Cell proliferation, S and G2/M phases, p-p21, pAkt	[154]
				↑ PDK4, p21Cip1, p-EGFR/MAPK	
	In vivo	TE-4 cells injected mice	Efatutazone	↓ Tumor growth, pAkt, p21, Ki67	[154]
				↑ PLIN2, p21Cip1, pEGFR, pERK1/2	
	In vitro	EC109 and TE10	Cisplatin (DDP)	↑ Apoptosis, Bax	[133]
				↓ Cell viability, Colony formation, Bcl-2	
	In vivo	Female BALB/C nude mice	DDP	↓ Tumor volume, Tumor weight	[133]
	In vivo	NMBzA induced F344 rats	Lycopene	↑ PPAR*γ*, Cleaved caspase 3 ↓ NF-*κ*B, COX-2	[155]
	In vitro	Eca-109	Da-Ea	↑ PPAR*γ*, Morphological alterations, Apoptosis, S phase cell cycle arrest	[97]
				↓ Cell proliferation	
	In vitro	OE33, ESO26	T0070907	↓ PPAR*γ*, Cell proliferation, Colony growth ↑ Apoptosis	[156]
	In vivo	Nude mice (ESO26 cells) xenografts	T0070907	↓ Tumor growth, FASN, ACC, ACLY, SCD	[156]
PPAR*γ*/RXR*α*	In vitro	KYSE series	Troglitazone +	↓ Cell growth	[157]
			9-cis retinoic acid	↑ G1 arrest, Apoptosis, Cleaved PARP	
	In vivo	KYSE 270 cells injected Balb/c-nu/nu mice	Troglitazone + 9-cis retinoic acid	↓ Tumor growth	[157]
PXR	In vitro	OE19, HET1A	Lithocholic acid	↑ Nuclear translocation of PXR protein levels	[134]
RAR*α*	In vitro	TE-10 and Eca-109	siRNA	↓ RAR*α*, Cell proliferation, Invasion, Migration, PCNA, Ki67, MMP7, MMP9, P-Glycoprotein, Wnt/*β*-catenin pathway activation, p-GSK3*β*^Ser9^, Cell viability ↑ p-GSK3*β*^Tyr216^, p-*β*-catenin^Ser33/37^, Susceptibility to 5-FU or CDDP	[136]
RAR*β*	In vitro	HET-1A, TE-3, TE-12	BPDE	↓ RAR*β*, G1 phase	[158]
				↑ COX-2, S phase	
		HET-1A, TE-3, TE-12	ATRA	↑ RAR*β*, G1 phase	
				↓ S phase	
	In vitro	TE-3 cell line	13-cis RA	↑ RAR*β*, Apoptosis	[159]
			AGN193109	↓ Cell growth, COX-2, Prostaglandin E2	
				↑ COX-2	
	In vitro	KYSE30 and TE-1	5-Aza-2-dC	↑ RAR*β*	[138]
		Het-1A	NNK	↑ Cell proliferation, DNMT1	
				↓ RAR*β*, Apoptosis	
RAR*β*2	In vitro	EC109	4HPR	↓ Cell growth	[160]
				↑ RAR*β*2, G0/G1 phase cell cycle arrest, Apoptosis	
			RA	↑ RAR*β*2	
	In vitro	KYSE4103	5-aza-dc	↑ RAR*β*2	[140]
	In vitro	KYSE150, KYSE410	5-aza-dc	↑ RAR*β*2 ↓Cell growth	[139]
	In vitro	TE-1, TE-8 TE-3-V1	BPDE BPDE	↓ RAR*β*2 ↑ EGFR, pERK1/2, COX-2, c-Jun, AP-1 ↑ COX-2	[161]
	In vivo	nu/nu nude mice (RAR*β*2 antisense-transfected TE3-A5cells)		↑ Tumor growth, EGFR, pERK1/2, COX-2	[161]
	In vitro	TE-1, TE-3, TE-8, TE-12	13-cis RA	↑ RAR*β*2	[162]
				↓ COX-2	
	In vivo	nu/nu nude mice (RAR*β*2 transfected TE-8 and sub cell lines)		↑ COX-2 ↓ Tumor development	[162]
	In vitro	TE-3, TE-12, SKGT4	BPDE	↓ RAR*β*2	[163]
				↑ c-Jun, pERK1/2, COX-2	
	In vitro	TE-3, TE-8, HCE-4, SKGT-4	4-NQO	↓ RAR*β*2	[164]
				↑ p-ERK1/2, c-FOS, COX-2	
	In vivo	4-NQO induced C57LB6/129Sv mice		↓ RAR*β*2 ↑ Tumor formation, p-ERK1/2, COX-2	[164]
RAR*γ*1	In vitro	EC109	4HPR, RA	↑ RAR*γ*1	[160]

↑—Increase/Upregulation; ↓—Decrease/Downregulation.

**Table 3:** Mechanistic role of various nuclear receptors (NRs) in esophageal cancer in clinical studies.

Nuclear Receptors (NRs)	Model/Cell Lines	Results	References
AR	EAC tissues	↓ AR	[165]
	EAC tissues	↑ AR, FKBP5	[147]
	ESCC tissues	↑ AR, Tumor progression	[119]
	ESCC tissues	↑ AR, Tumor growth, Tumor invasion Overexpressed AR leads to poor prognosis	[120]
ER*α*	ESCC tissues	ER*α* positive leads to lymph node metastasis, venous invasion and poor survival	[166]
	ESCC tissues	Absence of ER*α* leads to poor prognosis	[121]
	ESCC tissues	ER*α* negative favors better prognosis	[167]
ER*β*	EAC tissues	↑ ER-B isoforms	[124]
	ESCC tissues	ER*β* negative leads to poor survival	[166]
	EAC and ESCC tissues	↑ ER*β*, Dedifferentiation, Tumor stage	[125]
	Thoracic ESCC tissues	↑ ER*β*, Tumor differentiation, Ki67/MIB1 LI, Poor survival	[123]
	ESCC tissues	↑ ER*β*, Poor prognosis	[121]
	ESCC tissues	ER*β* positive favors better survival	[167]
FXR	EAC tissues	↑ FXR, Tumor grade, Tumor size, Lymph node metastasis ↓ RAR*β*2	[64]
	GERD tissues	↑ FXR, TLR2 ↓ TLR4	[128]
PPAR*γ*	ESCC tissues	↑ SIRT1, PPAR*γ*, EGFR, Survivin	[168]
	EC tissues	↓ PPAR*γ*	[133]
RAR*α*	EC tissues	↑ RAR*α,* Metastasis	[136]
RAR*β*	EC tissues	↓ RAR*β*	[169]
	EC tissues	↓ RAR*β* ↑ p53, Ki67	[170]
	ESCC tissues	↓ RAR*β*, CRBP1, TIG1 ↑ Tumor stage	[171]
	ESCC tissues	↓ RAR*β* ↑ Metastasis	[172]
	ESCC tissues	↓ RAR*β* ↑ DNMT1	[138]
RAR*β*1	ESCC tissues	↓ RAR*β*1 ↑ Cyclin D1, EGFR	[141]
RAR*β*2	ESCC tissues	↑ Methylation of RAR*β*2, p16, MGMT, CLDN3, CRBP, MT1G	[173]
	EC tissue	↑ RAR*β*2 ↓ COX-2	[162]
	EC tissues	↑ p-ERK1/2, COX-2, Tumor de-differentiation	[164]
	ESCC tissues	↓ RAR*β*2, LINE-1 ↑ Metastasis	[174]
	ESCC tissues	↓ RAR*β*2, COUP-TFI, COUP-TFII	[141]
RAR*β*4	ESCC tissues	↓ COUP-TFI, COUP-TFII ↑ RAR*β*4	[141]
RXR*α*	EAC tissues	↓ RXR*α*	[175]
	EC tissues	↑ RXR*α*, TNM stage, Metastasis	[176]
RXR*β*	EAC tissues	↓ RXR*β*	[175]
	ESCC tissues	↓ RXR*β* ↑ Metastasis	[172]
RXR*γ*	EAC tissues	↑ RXR*γ*	[175]
VDR	BE tissues	↑ VDR	[177]
	EAC tissues	↓ VDR, Tumor de-differentiation	[143]
	EAC and ESCC tissues	↑ VDR, TGR5, Claudin-2	[178]
	EAC tissues	↓ VDR, CYP27B1 ↑ CYP24A1	[179]

↑—Increase/Upregulation; ↓—Decrease/Downregulation.

## Data Availability

Not applicable.

## Abbreviations

5-aza-dc-5-aza-20	deoxycytidine
4HPR	*N*-(4-hydroxyphenyl) retinamide
AC	Adenocarcinoma
AF	Activation function
AR	Androgen receptors
ARE	Androgen Responsive Elements
ATRA	All-trans retinoic acid
BA	Bile acid
BE	Barrett’s esophagus
BPDE	Benzo-[a]pyrene diol epoxide
CCM	Columnar cell metaplasia
CCK2	Cholecystokinin 2
CDDP	Cisplatin, cisplatinum, or *cis*-diamminedichloroplatinum (II)
COUP TF	Chicken ovalbumin upstream promoter transcription factor
COX-2	Cyclooxygenase-2
CRBP-1	Cellular retinol-binding protein-1
CTD	C-terminal domain
Da-Ea	Ethyl acetate extract of *D. altaica*
DBD	DNA-binding domain
DDP	Diamminedichloroplatinum
DFS	Disease-free survival
DHT	Dihydrotestosterone
DNMT1	DNA (cytosine-5)-methyltransferase1
DNMT3A	DNA (cytosine-5)-methyltransferase 3A
DPN	Diarylpropionitrile
E2	17*β*-estradiol
EAC	Esophageal adenocarcinoma
EC	Esophageal cancer
EGFR	Epidermal growth factor receptor
ER	Estrogen receptor
EREs	Estrogen response elements
ERK	Extracellular-signal regulated kinase
ERRs	Estrogen related receptors
ESCC	Esophageal squamous cell cancer
ESR	Estrogen receptor
FAK	Focal adhesion kinase
FBXO32	F-Box Protein 32
FDA	Food and drug administration
FGFR	Fibroblast growth factor receptors
FKBP5	FK506-binding protein 5
FU	Fluorouracil
FXR	Farnesoid X receptor
GCA	Gastric cardia adenocarcinoma
GERD	Gastroesophageal reflux disease
GRs	Glucocorticoid receptors
HGF	Hepatocyte growth factor
HMOX1	Heme Oxygenase 1
HREs	Hormone response elements
HSP90	Heat shock protein 90
IL-6	Interleukin-6
JNK	c-Jun N-terminal kinases
KLK3	Kallikrein Related Peptidase 3
LBD	Ligand binding domain
LCA	Lithocholic acid
LDH	Lactate dehydrogenase
LINE-1	Long Interspersed Element-1
LXRs	Liver X receptor
MAPK	Mitogen-activated protein kinase
MMP	Matrix metalloproteinase
MPP	Methyl-piperidinopyrazole
MRTF	Master Regulator Transcription Factors
NCoR	Nuclear receptor corepressor
NF-κB	Nuclear factor kappa B
NNK	4-(*N*-methyl-*N*-nitrosamino)-1-(3-pyridyl)-1-butanone
NQO	Nitroquinoline 1-oxide
NRs	Nuclear receptors
NR1H4	Nuclear Receptor Subfamily 1 Group H Member 4
NR4A1	Nuclear receptor 4A1
NTD	*N*-terminal domain
OS	Overall survival
PAR	Pregnane-activated receptor
PCNA	Proliferating cell nuclear antigen
PG	Prostaglandin
PGC-1	PPAR coactivator-1
PHTPP	4-[2-Phenyl-5,7-bis(trifluoromethyl)pyrazolo[1,5-a]pyrimidin-3-yl]phenol
PLIN2	Perilipin 2
PPAR	Peroxisome Proliferator Activated Receptor
PPRE	Peroxisome proliferator response element
PPT	1,3,5-tris(4-hydroxyphenyl)-4-propyl-1*H*-pyrazole
PRs	Progesterone receptors
PXR	Pregnane X receptor
RA	Retinoic acid
RAR	Retinoic acid receptor
RAREs	Retinoic acid receptor elements
RRIG1	Retinoid receptor-induced gene-1
RXR	Retinoid X receptor
SERMs	Selective estrogen receptor modulators
SCC	Squamous cell carcinoma
SIRT1	Sirtulin 1
SMRT	Silencing mediator of retinoic acid and thyroid hormone receptor
STAT3	Signal Transducer and Activator of Transcription 3
SXR	Steroid and xenobiotic receptor
TFs	Transcription factors
TGR5	Takeda G protein-coupled receptor 5
TIG1	Tazarotene-induced gene-1
TLRs	Toll-like receptors
TNM	Tumor nodes metastases
TR	Thyroid hormone receptor
TRO	Troglitazone
TZD	Thiazolidinedione
VDR	Vitamin D receptor
VDR	Vitamin D receptor elements
VEGFA	Vascular endothelial growth factor A
WNT5A	Wnt Family Member 5A
